# Inhibitory framing in hypersexual patients with Parkinson’s disease. An fMRI pilot study

**DOI:** 10.1007/s00221-022-06397-5

**Published:** 2022-06-28

**Authors:** Hendrik Theis, Catharina Probst, Anna Campabadal, Katharina S. Goerlich, Oliver Granert, Stephan Wolff, Karsten Witt, Günther Deuschl, Thilo van Eimeren

**Affiliations:** 1 Faculty of Medicine, Department of Nuclear Medicine, University of Cologne, Cologne, Germany; 2 Faculty of Medicine, Department of Neurology, University of Cologne, Cologne, Germany; 3 Faculty of Medicine, Medical Psychology Unit, Department of Medicine, University of Barcelona, Barcelona, Spain; 4 Department of Biomedical Sciences of Cells & Systems, Section Cognitive Neuroscience, University Medical Center Groningen, University of Groningen, Groningen, The Netherlands; 5 Faculty of Medicine, Department of Neurology, University of Kiel, Kiel, Germany; 6 Faculty of Medicine, Department of Radiology, University of Kiel, Kiel, Germany; 7 Carl von Ossietzky‐University, Department of Neurology, Evangelical Hospital Oldenburg and Research Center of Neurosensory Sciences, Oldenburg, Germany

**Keywords:** Addiction, Functional MRI, Impulse control, Dopamine

## Abstract

Hypersexuality in medicated patients with PD is caused by an increased influence of motivational drive areas and a decreased influence of inhibitory control areas due to dopaminergic medication. In this pilot study, we test a newly developed paradigm investigating the influence of dopaminergic medication on brain activation elicited by sexual pictures with and without inhibitory contextual framing. Twenty PD patients with and without hypersexuality were examined with fMRI either OFF or ON standardized dopaminergic medication. The paradigm consisted of a priming phase where either a neutral context or an inhibitory context was presented. This priming phase was either followed by a sexual or a neutral target. Sexual, compared to neutral pictures resulted in a BOLD activation of various brain regions implicated in sexual processing. Hypersexual PD patients showed increased activity compared to PD controls in these regions. There was no relevant effect of medication between the two groups. The inhibitory context elicited less activation in inhibition-related areas in hypersexual PD, but had no influence on the perception of sexual cues. The paradigm partially worked: reactivity of motivational brain areas to sexual cues was increased in hypersexual PD and inhibitory contextual framing lead to decreased activation of inhibitory control areas in PD. We could not find a medication effect and the length of the inhibitory stimulus was not optimal to suppress reactivity to sexual cues. Our data provide new insights into the mechanisms of hypersexuality and warrant a replication with a greater cohort and an optimized stimulus length in the future.

## Introduction

Hypersexuality (HS), or compulsive sexual behavior is one of the most frequent disorder of the spectrum of impulsive-compulsive behaviors (ICBs) associated with dopaminergic treatment in Parkinson’s disease (PD) (Nakum and Cavanna 2016). The pathophysiology underpinning HS and other ICBs is currently seen as largely similar to substance addiction, which is why these behaviors are also commonly referred to as behavioral addictions (ICD-11 for Mortality and Morbidity Statistics 2022; Falkai et al. 2020). Based on the sensitization theory of addiction, ICBs form as a consequence of conditioning via the dopaminergic reinforcement system (sometimes referred to as “reward system”), to which the ventral striatum is the key player (Robinson and Berridge 1993). As a consequence, a habitual cue-response pattern (i.e., positive emotional association bias towards addiction cues) and an insufficient inhibitory control are observed in ICBs (Potenza 2008; Probst and van Eimeren 2013).

As recently proposed, the development of ICBs in PD is linked to a combination of a premorbid predisposition to sensitization and a relative dopaminergic overdosing both involving the ventral striatum (Theis et al. 2021). And indeed, an increased reactivity of motivational drive areas to addiction cues, and a deficient neuronal recruitment of inhibitory control areas have both been described in PD patients who had developed an ICB (Cilia and van Eimeren 2011). A key feature of ICB is an impaired inhibitory control (Probst and van Eimeren 2013). Inhibitory control is the ability to suppress undesired or inappropriate actions, which largely overlaps with response inhibition (Criaud et al. 2021). The term “inhibitory control” is often used as an umbrella term in psychology describing the objectively and subjectively perceived ability to suppress behavioral responses or thoughts, particularly when the lack of control is associated with negative long-term consequences. In contrast, the term “response inhibition” is more focused and often used psychometrically to describe the suppression of a (prepotent) motor response. Several neuroimaging studies examined response inhibition in healthy participants and PD patients with ICB with paradigms that do not involve reward processing highlighting the involvement of similar regions, such as the ventral striatum and cingulate cortex (Criaud et al. 2017, 2021; Meyer et al. 2020). However, how the dynamic interplay of inhibitory context and cue-reactivity is changed following dopaminergic stimulation is not well understood. A recent review on functional imaging in ICB advocates that imaging studies should use more elaborated psychological models and behavioral designs to adequately assess the underlying neurocognitive processes (Meyer et al. 2019). Therefore, our aim was to provide a neuroscientific basis to study the interaction of sexual cue processing and inhibitory control with functional MRI (fMRI) and chose HS as a frequent and well-defined form of ICB in PD.

A previous fMRI study examined the influence of sexual visual cues on hypersexual PD patients (PD+HS) and PD controls (PD-HS) as a function of dopaminergic medication (Politis et al. 2013). The authors found an increased activation in the ventral striatum, the cingulate and orbitofrontal cortices in PD+HS due to sexual visual cues. Activations in similar regions were found in a delay discounting task with sexual stimuli (Girard et al. 2019). We wanted to optimize the fMRI protocol of Politis et al. for our purpose by adding an inhibitory contextual framing. In healthy individuals, negative mood states such as disgust have been found to generally suppress sexual arousal through increased inhibitory brain activity (Koukounas and McCabe 2001; Bradford and Meston 2006; Janssen et al. 2008; Andrews et al. 2015). Hence, we reasoned that aversive images associated with skin disease preceding the sexual cues may provide a contextual inhibitory framing modulating the response to sexual cues.

This exploratory study had multiple objectives: (i) to replicate earlier results obtained without inhibitory framing; (ii) to confirm that the inhibitory framing would engage inhibitory brain regions and be associated with a subsequent reduction of reward-related activity following sexual cues; (iii) to probe the validity of our hypothesis, that when comparing PD+HS to PD-HS, the inhibitory context would be less effective in suppressing excitability of motivational drive areas, particularly under dopaminergic medication.

## Methods

### Participants

The initial study sample consisted of 21 PD patients recruited from an outpatient movement disorders clinic (Department of Neurology, University Hospital Kiel). The inclusion criteria for PD were: (1) the fulfillment of UK PD Society Brain Bank diagnostic criteria for PD; (2) no treatment with deep brain stimulation. Exclusion criteria were: (1) Montreal Cognitive Assessment scores < 21 (Marinus et al. 2011), (2) MRI contraindications; (3) pathological MRI findings other than mild white matter hyperintensities. Patients were recruited from a larger sample originating from a previous prospective study (Probst et al. 2014). All study procedures followed the declaration of Helsinki and the study was approved by the University of Kiel Ethics Committee. Participants received 8 Euros per hour as partial compensation for their time. The final study sample consisted of 20 patients after excluding one participant due to severe cortical atrophy. Three patients fell asleep during one of the sessions, two of them agreed to repeat the session. We excluded from the analysis those sessions patients fell asleep in, as well as one session that was lost for technical reasons.

### Clinical assessment

All PD patients were taking antiparkinsonian medication consisting of different combinations of L-DOPA, COMT inhibitors, MAO inhibitors, dopamine agonists, and amantadine. We calculated the levodopa equivalent dose and levodopa equivalence dose for dopamine agonists following previous methods (Goerlich-Dobre et al. 2014). Functional MRI (fMRI) sessions were done under medication and without medication intake.

### ICB diagnosis

All patients underwent a clinical diagnostic interview conducted by a trained psychologist (C.P.) who assessed the criteria of depression, mania and ICB. A detailed screening of hypersexuality, pathological gambling, excessive buying, binge eating, punding, hobbyism and Dopaminergic Dysregulation Syndrome was done (Probst et al. 2014). In addition, patients completed self-report questionnaires assessing the presence of ICBs: the 125-item version of the Temperament and Character Inventory (Berth et al. 2001), the Barratt Impulsiveness Scale (Patton et al. 1995; Preuss et al. 2008), the Emotion Regulation Questionnaire (Gross and John 2003; Abler and Kessler 2009), the Sexual Inhibition/Excitation Scales (Janssen et al. 2002; Carpenter et al. 2008), the Behavioral Inhibition/Activation Scales (Carver and White 1994; Strobel et al. 1999), the Bermond-Vorst Alexithymia Questionnaire (Vorst and Bermond 1999), the Beck Depression Inventory (BDI-II) (Hautzinger et al. 2009), the Beck Anxiety Inventory (Margraf and Ehlers 2007) and the Questionnaire for Impulsive-Compulsive Disorders-Rating Scale (Weintraub et al. 2012; Probst et al. 2014).

We carried out two-sample *t*-tests to characterize potential group differences in demographic and clinical data (*p* < 0.05 was considered significant).

### Procedure and paradigm

Patients were examined on two different days during the afternoon, with at least 24 h in between the measurements. Before the sessions, participants had to refrain from taking their antiparkinsonian medication for at least 24 h. Extended-release medication was paused at least 72 h before the appointments. Participants were scanned ON and OFF medication (sessions were counterbalanced). Adapted from (Politis et al. 2013) and (van Eimeren et al. 2009), participants took 12.5 mg/50 mg tablets of Carbidopa/Levodopa and 1.05 mg Pramipexol about 50 min before scanning in the ON condition to standardize the medication effect. This dose was chosen as a pragmatic combination of levodopa and dopamine agonist effects, since both types of medication play significant roles in ICB development (Weintraub et al. 2010). Patients were told to avoid eating at least 1 hour before the ON condition. The presence of motor symptoms was evaluated using the Unified Parkinson’s Disease Rating Scale motor section (UPDRS III) in the OFF session (UPDRS III-OFF_1_), and twice in the ON session, right before medication (UPDRS III-OFF_2_) and 40 min after medication intake (UPDRS III-ON_2_).

**Fig. 1:**
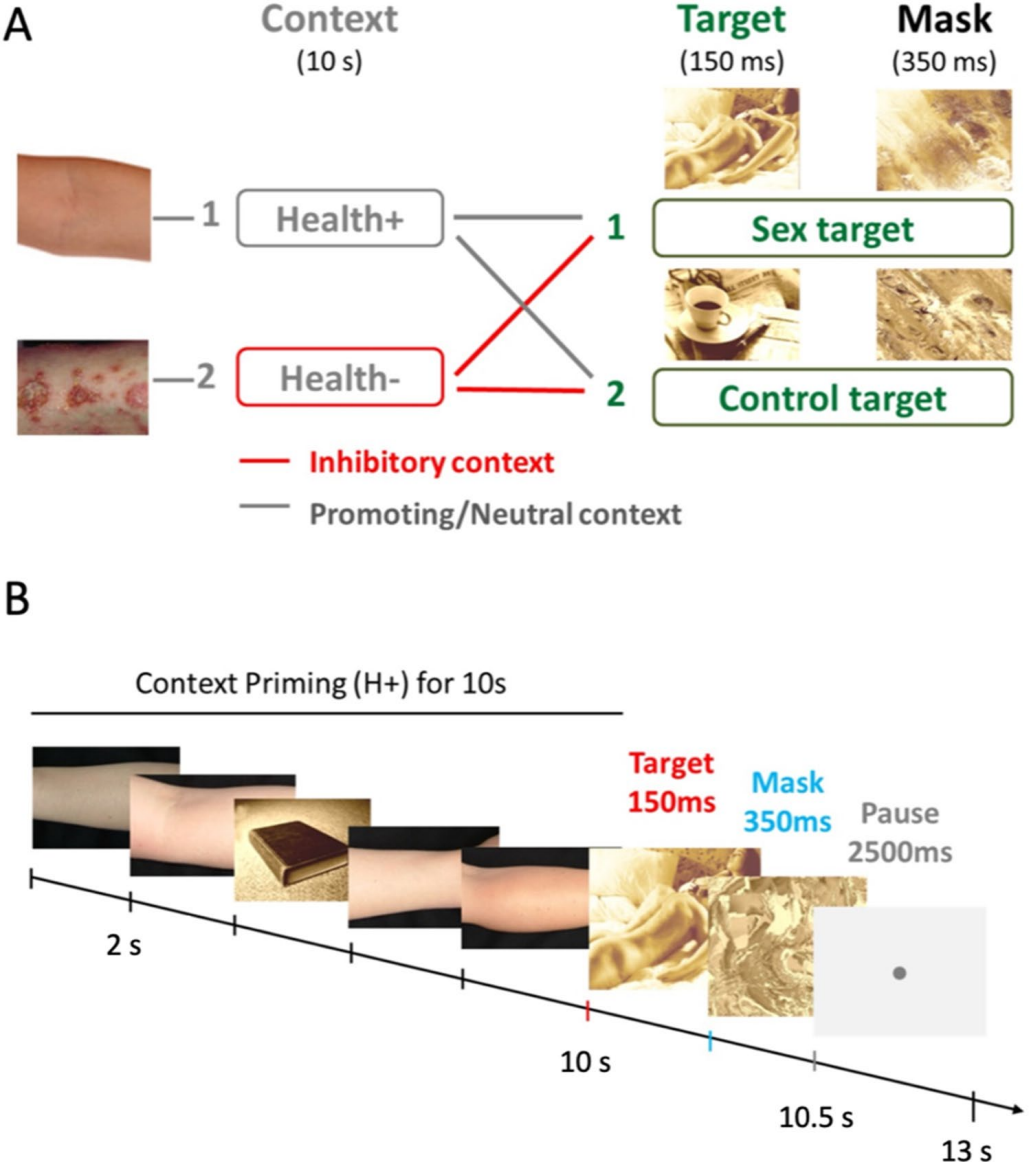
**A** Paradigm structure—Four, five or six consecutive pictures of either healthy skin (neutral context) or of infectious skin (inhibitory context) were followed by a sexual target or a neutral control target. Both were masked with a scrambled version. Each context–target combination was shown ten times per session and participants watched three sessions. **B** Timing of one paradigm block. During the context priming skin pictures were shown for 2 s each. In eight blocks of each session, one of the skin pictures was replaced with a book (attention check). The target was displayed for 150 ms and followed by a mask (350 ms). The jittered pause lasted on average 2500 ms

During scanning, patients participated in an fMRI paradigm (programmed in E-prime^®^ 1.2, Psychology Software Tools, Inc.) consisting of three sessions. The paradigm comprised priming blocks of four, five or six pictures (equally frequent) of healthy (H+ , neutral context) or infectious skin (H-, inhibitory context) that were displayed one after another and followed by either a sexual target or a neutral control cue. We used pictures with infectious skin diseases since these are associated with disgust and are known to repress sexual arousal (Curtis and Biran 2001; Andrews et al. 2015). Figure 1 depicts the paradigm structure and timing of one block. Each session lasted about 9.6 min and consisted of 40 blocks (20 blocks of H + context and 20 of H- context, in pseudorandom order). Each context block was followed by a either a sexual target or a control cue (balanced, pseudorandom). For more details regarding the paradigm structure, please consult Fig. 1B. We opted to use brief, yet supra-threshold visual presentation times for target and control cue, since subliminal stimulus times similar to Andrews et al. did not lead to a reliable activation of brain activation in PD patients in prior pilot experiments by us (Andrews et al. 2015). As a check for attention, in eight blocks of each session, a book was shown instead of one of the first three priming pictures, and participants had to press a mouse key to indicate that they saw the book. The sexual pictures were chosen from the International Affective Picture System (Lang et al. 2008). Sexual and neutral pictures were displayed in sepia color to increase perceptual similarity.

### MRI acquisition and preprocessing

MRI were acquired with a 3T scanner (Achieva; Philips, Best, the Netherlands) equipped with a 32-channel head coil. The scanning protocol included 101 T2*-weighted whole brain echo planar images (EPI) per session [TR = 2500 ms, TE = 33 ms, flip angle = 90°, FOV = 210 × 210 mm, slices per EPI = 35; slice thickness = 3 mm, inter-slice gap = 0.3 mm]. T1- and T2-weighted structural images were also acquired for each patient. During the scanning, participants wore earplugs as well as headphones. Stimuli were projected to a screen and participants saw the images via a mirror attached to the head coil. After half of the participants were scanned with this procedure (equal number of PD+HS and PD-HS), an MRI-compatible VisualSystem from NordicNeuroLab with integrated vision correction was used for stimulus presentation both with 800 × 600 pixel resolution.

The fMRI datasets were preprocessed and analyzed using SPM8 software (Wellcome Department of Cognitive Neurology, London, UK) implemented in MATLAB (Mathworks) as follows: EPI images were realigned to correct for head motion. Subsequently, the T1 image was normalized using the SPM-segmentation algorithm. This algorithm includes an intensity normalization procedure to compensate for magnetic inhomogeneities. Then the EPI images were co-registered to the intensity corrected T1 image and normalized using the parameter estimates from the segmentation algorithm. The normalized fMRI images were spatially smoothed with a Gaussian kernel of 8 mm (full-width at half-maximum).

The first-level was set up separately for each participant and session (ON, OFF), leading to two first-level models per participant. We set up a general linear model including the priming context as a block of 10 s and sexual and neutral cues as two event types (duration 0). Button presses (= book pictures) were also modeled. Movement parameters from realignment were added as multiple regressors. Contrasts were set up as follows: Context H+  > Context H, Context H- > Context H+, sexual > neutral target, (sexual > neutral) for Context H+ and (sexual > neutral) for Context H-. On the second level, we set up a flexible factorial model with the contrast images ‘sexual > neutral target’ and with subject as random factor. Fixed factors were group (PD+HS, PD-HS), context (H-, H+) and medication status (ON, OFF). First, we looked at the overall effect of target versus control pictures for all participants. Second, we specifically analyzed the effects of the fixed factors group, context and medication. We finally examined the various interactions of group, context and medication. Due to the exploratory nature of this study, we report results at a significance level of *p* < 0.001, uncorrected, with a cluster size > 10 voxels.

## Results

### Clinical and behavioral characteristics

Ten PD+HS were diagnosed with active and at least subsyndromal symptoms (Probst et al. 2014) of a hypersexual disorder according to proposed criteria (Voon and Fox 2007). Most PD+HS patients presented, in addition to HS, other subsyndromal ICBs [excessive buying (*n* = 2); overeating (*n* = 1); and punding (*n* = 1)]. Ten PD-HS were included. One PD-HS patient used to have slight overeating problems decades before PD onset. Three PD-HS and one PD+HS patients had at least subsyndromal symptoms of depression according to the diagnostic interview. Nineteen patients reported to be heterosexual and one PD+HS patient reported to be bisexual.

Groups did not differ significantly in sex, age, antiparkinsonian doses or disease duration (Table 1). PD+HS patients had higher scores than PD-HS in the UPDRS III-OFF_2_ (*t* = − 2.59; *p* < 0.05). Moreover, UPDRS III-ON_2_ was increased by 23.6% in the PD-HS and 26.2% in PD+HS compared to the OFF condition (UPDRS III-OFF_2_). Table 1 shows those questionnaires data that were significantly different between PD+HS and PD-HS.

**Table 1:** Demographical, clinical and ICD measures of Parkinson’s disease patients

	PD+HS (*n* = 10)	PD-HS (*n* = 10)	*t*-value
Age	56.2 (12.37)	60.10 (7.92)	NS
Sex (male/female)	(8/2)	(8/2)	NS
PD duration	6.05 (4.52)	7.15 (5.46)	NS
UPDRS III-OFF_1_	30.25 (10.4)	22.9 (8.36)	NS
UPDRS III-OFF_2_	33.25 (9.51)	22.5 (8.13)	-2.59**
UPDRS III-ON_2_	22.56 (11.48)	17.2 (8.44)	NS
LED	866.69 (532.14)	814.34 (682.37)	NS
LEDDA	237.45 (249.99)	312.19 (209.01)	NS
MoCA	27.7 (1.5)	26.7 (1.16)	NS
**Group comparison for ICD measures**			
BDI-II	9.4 (4.79)	19.9 (13.08)	2.38**
BVAQ Fantasizing	23.44 (2.74)	27.6 (4.86)	2.26*
BAS Sum-score	35.33 (10.78)	27.0 (7.44)	-1.98*
BAS Drive	13.78 (3.8)	9.9 (2.88)	-2.52*
BAS Reward	13.56 (4.5)	9.6 (3.5)	-2.15*
QUIP-RS Sex	9.8 (4.64)	5.78 (4.24)	-1.97*

Measures are presented as mean (standard deviation) and test-statistics (*t*-value)

*BAS Sum-score* the behavioral activation scale total score, *BAS Drive* the behavioral activation scale drive score, *BAS Reward* the behavioral activation scale reward responsiveness score, *BDI-II* beck depression inventory-second edition, *BVAQ* the bermond-vorst alexithymia questionnaire fantasizing subscale, *LED* levodopa equivalence doses, *LEDDA* levodopa equivalence dose for dopamine agonists, *MoCA* montreal cognitive assessment, *NS* non-significant result, *PD* Parkinson’s disease; *PD*+*HS* PD with hypersexuality, *PD-HS* PD without hypersexuality, *QUIP-RS Sex* questionnaire for impulsive-compulsive disorders-rating scale, Sex subscale. *UPDRS III* unified Parkinson’s disease rating scale motor section, *UPDRS III-ON*_2_ UPDRS III for the ON condition 40 min after medication intake, *UPDRS III-OFF*_1_, UPDRS III for the OFF condition, *UPDRS III-OFF*_2_ for the ON condition before taking medication. **p < 0.05 (two-sided). *p < 0.05 (one-sided). Only those ICD measures showing statistical significance between groups are included

### Functional MRI results

All the analyses presented in the following sections report on sexual cue specific activation (sexual vs. neutral cue). Bear in mind that the results of this exploratory study are reported with a relatively liberal threshold for type I errors (*p* < 0.001 uncorrected, cluster size > 10).

### Main effect of target: sexual vs control

**Fig. 2:**
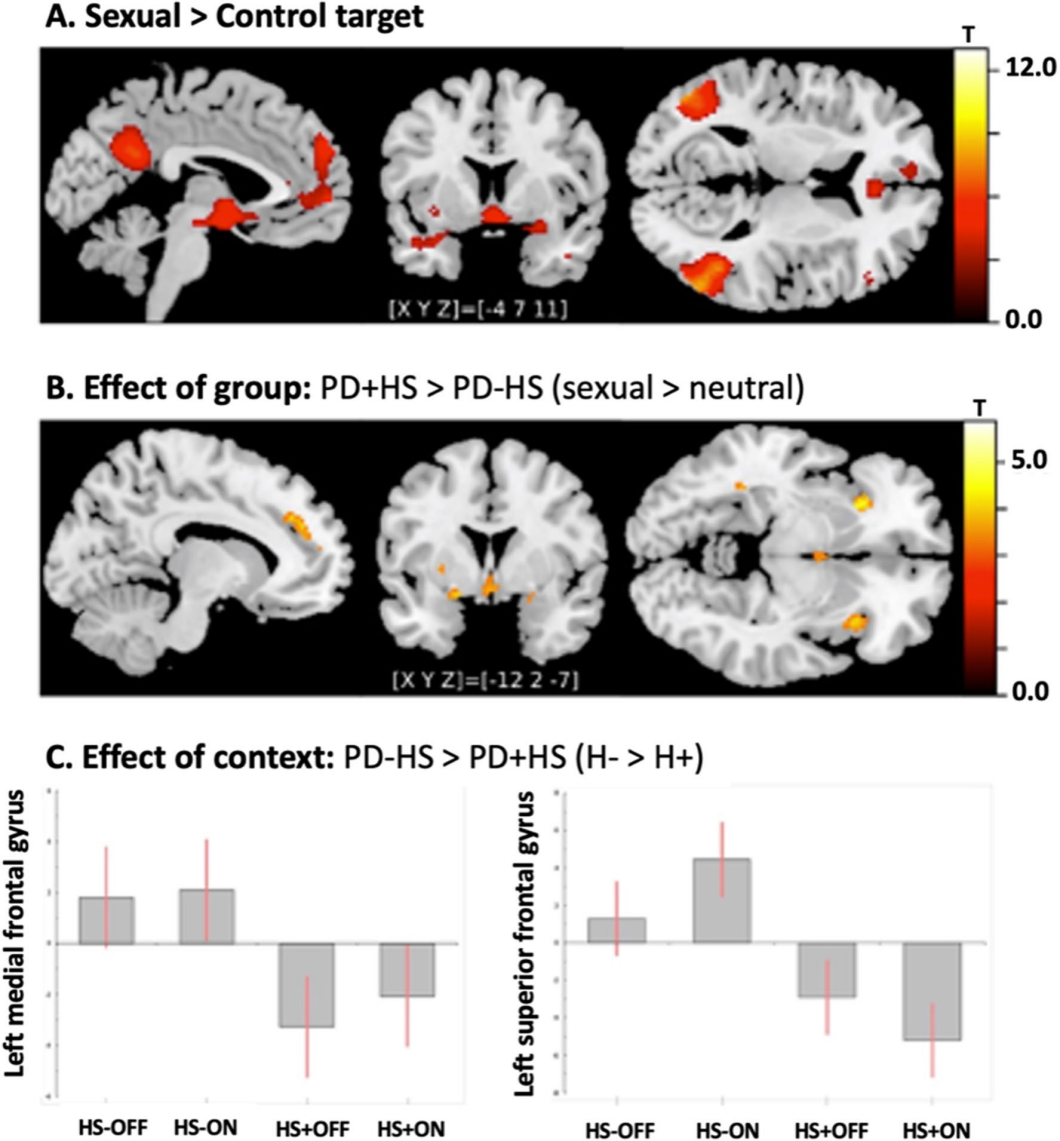
Functional MRI results **A** Brain regions with increased activity in sexual versus control pictures (contrast for the whole sample, including all conditions), **B** increase activation in PD+HS relative to PD-HS when seeing sexual targets in comparison to control cues, **C** the bars represent increased activation during inhibitory context (H-) relative to non-inhibitory context (H+) for the peak voxels of the two significant clusters in the left medial frontal gyrus (left panel) and left superior frontal gyrus (right panel). H + neutral context, H- Inhibitory context, HS+ Hypersexual patients, HS- non-hypersexual patients, OFF/ON is referring to medication status, *PD* Parkinson’s disease. Color bars represent *t*-value

Sexual in comparison to neutral cues elicited activation amongst others in the following brain regions: bilateral parahippocampus, amygdala, hypothalamus, frontal, temporal and occipital, as well as the midbrain (Table 2 and Fig. 2A).

**Table 2:** Brain regions showing higher activity in sexual targets in comparison to control cues

Brain region	MNI			*t-*value
	*x*	*y*	*z*	
L Amygdala	− 16	− 4	− 20	4.1
L Fusiform gyrus	− 42	− 54	− 16	10.45
L Hypothalamus	− 4	− 2	− 12	5.94
L Inferior Frontal gyrus (Pars Triangularis)	− 48	32	2	4.06
L Insula	− 26	16	− 16	5.96
L Lateral orbitofrontal gyrus	− 32	28	− 14	3.47
L Middle occipital gyrus	− 44	− 74	18	6.17
L Middle temporal gyrus	− 48	− 70	10	8.31
L Parahippocampal gyrus	− 20	− 26	− 16	3.54
L Posterior cingulate	− 4	− 52	26	6.68
L Superior frontal gyrus	− 4	60	0	5.21
L Superior medial Frontal gyrus	− 8	58	32	6.07
Midbrain	− 2	− 16	− 12	4.52
R Amygdala	26	2	− 18	5.03
R Cerebellum	6	− 54	− 2	3.27
R Fusiform gyrus	42	− 48	− 16	13.04
R Hippocampus	34	− 20	− 16	3.42
R Hypothalamus	6	0	− 8	7.5
R Inferior frontal gyrus (pars triangularis)	52	34	14	3.46
R Inferior occipital gyrus	46	− 74	− 4	8.43
R Lateral orbitofrontal gyrus	32	30	− 16	3.89
R Middle pole of middle temporal gyrus	44	8	− 32	3.87
R Middle temporal gyrus	56	− 62	10	8.37
R Middle occipital gyrus	46	− 72	4	7.5
R Parahippocampal gyrus	22	− 22	− 18	3.55
R Rostral anterior cingulate cortex	2	36	12	4.55
R Superior frontal gyrus	10	60	34	3.33

This contrast included the collapsed PD sample and all experimental conditions. Coordinates are in MNI space

### Effect of group

The contrast sexual versus neutral target elicited more activation in PD+HS than in PD-HS involving amongst others the following brain areas: bilateral fusiform gyrus, anterior insula, amygdala, superior frontal gyrus, left putamen and thalamus (Table 3 and Fig. 2B). On the other hand, PD-HS had more activation than PD+HS in left posterior middle temporal gyrus ([*x* *y* *z*] = [− 50 − 54 10]) during the presentation of sexual targets.

**Table 3:** Brain regions with increased activity in PD+HS relative to PD-HS when confronted to sexual targets in comparison to control cues

Brain region	MNI coordinates			*t* value
	x	y	z	
L Amygdala	− 20	2	− 12	4.1
L Fusiform gyrus	− 40	− 50	− 16	3.96
L Hypothalamus/ventricle	0	0	− 8	3.75
L Insula	− 30	24	− 4	4.74
L Putamen	− 26	4	4	3.41
L Superior frontal gyrus	− 12	38	32	4.33
L Thalamus	− 2	− 6	0	3.7
R Amygdala	22	6	− 16	4.11
R Fusiform gyrus	40	− 50	− 16	5.89
R Inferior frontal gyrus (Pars Triangularis)	32	22	16	3.64
R Inferior occipital gyrus	38	− 72	− 10	3.38
R Insula	36	20	− 4	3.97
R Middle frontal gyrus (dorsal prefrontal cortex)	38	46	10	3.37
R Superior frontal gyrus	10	46	30	3.87

Coordinates are in MNI space

### Effect of medication

#### ON vs. OFF

Increased activity during the ON compared to the OFF condition was detected in the left posterior cingulum ([*x* *y* *z*] = [− 8 – 42 − 10]), retrosplenial region), whereas higher activation was shown during the OFF relative to the ON session in the left anterior corona radiata ([*x* *y* *z*] = [− 20 28 − 6]), right lenticular fasciculus ([*x* *y* *z*] = [10 − 2 − 8]) and right middle frontal gyrus ([*x* *y* *z*] = [36 4 58]) while watching sexual versus neutral targets.

### Interaction of medication and group

When studying group differences related to antiparkinsonian medication, PD+HS had greater activation than PD-HS in the ON condition in right cerebellar crus ([*x* *y* *z*] = [10 − 78 − 28]). Contrast estimates showed that this effect was mainly driven by a deactivation in the ON state in PD-HS. The opposite contrast did not yield any significant results.

### Effect of context

When examining the influence of context (H+ versus H-; i.e., healthy vs. infectious looking skin) on the perception of sexual (versus neutral) targets, no significant results were obtained. Comparing the groups for H-> H+ revealed more activation in left medial frontal and left superior frontal gyri for PD-HS versus PD+HS. The contrast estimates revealed that in the medial frontal gyrus (Fig. 2C, left panel), relative hypoactivation in PD+HS was largely independent of medication, whereas in the superior frontal gyrus (Fig. 2C, right panel), the effect was mainly driven by an opposing trend in the ON state with an activation in the PD-HS group and a deactivation in PD+HS. We found no significant clusters when examining the subgroups separately for this contrast.

## Discussion

In this exploratory study, we tested a new paradigm containing reactivity to sexual cues with and without contextual inhibitory framing. We replicated the results of earlier studies showing a relatively increased reactivity of motivational areas to sexual cues in PD+HS, including the putamen and the superior frontal gyrus (Politis et al. 2013). During inhibitory framing in comparison to neutral framing (H-> H+), patients with HS demonstrated decreased activation of frontal regions involved in impulse control (left medial frontal and left superior frontal gyri) than patients without HS. However, we did not observe effects of the inhibitory contextual framing on reactivity to sexual cues.

Our exploratory approach allowed for a higher risk of false positive results and therefore, the results should be interpreted with great caution. Overall, we found increased reactivity to sexual cues in the PD+HS group. While there was no relevant effect of dopaminergic medication between the two groups. Inhibitory stimuli enhanced inhibitory activity and reduced reward activation in PD-HS but only marginally influenced reactivity in PD+HS. However, there was no significant effect of inhibitory context on the perception of sexual cues as compared to neutral cues.

In general, watching sexual compared to neutral stimuli in PD lead to a BOLD activation in the amygdala, the hypothalamus, the insula, the orbitofrontal, parahippocampal and fusiform gyri, anterior cingulate, as well as some in occipital regions. These areas have been systematically associated with erotic content processing (Arnow et al. 2002; Walter et al. 2008; Paul et al. 2008; Georgiadis and Kringelbach 2012; Wehrum-Osinsky et al. 2014) and are known to evaluate reward, incentive, emotional and arousing information (Georgiadis and Kringelbach 2012). Some of these regions form part of the salience network, which is disrupted in PD+HS (Navalpotro-Gomez et al. 2020). A recent study found enhanced activity in the salience network in PDHS as compared to PD-HS in resting-state fMRI. The authors conclude that these alterations might lead to an enhanced detection of sexual cues (Mata-Marín et al. 2021). In line with Politis et al*.,* our data confirmed increased activation in PD patients with HS in comparison to PD controls in such regions (Politis et al. 2013). Unfortunately, we could not find changes in the ventral striatum as previously described in neuroimaging studies about ICB (O’Sullivan et al. 2011; Politis et al. 2013; Voon et al. 2014). Recently, these changes have been proved in a first histopathological study which could detect lower levels of α-synuclein and D3 receptors in PD patients with ICB as compared to PD patients without ICB (Barbosa et al. 2019).

Regarding medication effects, PD patients showed heightened activity in the posterior cingulum in the ON relative to the OFF state while watching sexual cues. Whereas in the OFF state they had greater activity in the left anterior corona radiata, right lenticular fasciculus, and right middle frontal. In agreement with our finding, previous fMRI studies provide evidence for the impact of dopaminergic medication in brain activity in the posterior cingulate cortex during the viewing of sexual cues in hypersexual (Politis et al. 2013; Girard et al. 2019). In our study, dopaminergic medication did not have a different impact on PD+HS compared to PD controls in the classical impulsive control or reward/motivation regions, since we found only stronger activation in the right cerebellar crus in the ON versus the OFF state compared to PD-HS. While the cerebellum is not typically associated with ICB and we need to consider a false positive result, cerebellar abnormalities have been linked to compulsive symptomatology and behavioral inhibition in non-PD populations (Koehler et al. 2013; Narayanaswamy et al. 2016; Miquel et al. 2019). The sample size of this pilot study was small, which could be the most important reason for the missing results in typical ICB-related regions. Recruiting patients with hypersexuality was indeed very difficult, because many patients do not report hypersexuality to their neurologists and are ashamed of their behavior. Even less patients agree to participate in a study. In the future, a multi-center study would be more feasible to recruit a greater sample size. Furthermore, participants did not receive their individual dopaminergic medication and improvement in the UPDRS III under ON condition was relatively low particularly in the PD-HS group. So probably, not all patients might be in their individual ON state which could also be a reason for the weak medication effects.

As hypothesized, contextual inhibitory framing enhanced inhibitory activity in PD-HS and only marginally influenced inhibitory activity in PD+HS. The interaction of context with group revealed stronger activation in the left medial frontal and left superior frontal gyri in PD controls in comparison to hypersexual PD for inhibitory versus neutral context. Interestingly, inhibitory tasks not involving reward processing per se found that these regions are also important in response inhibition in healthy individuals and in PD patients with ICB (Criaud et al. 2017; Meyer et al. 2020). As described in the introduction, it is crucial to differentiate underlying deficits in response inhibition from difficulties to suppress behavior in a more general sense (Meyer et al. 2020). Importantly, the chosen paradigm may not definitely be ascribed to a specific concept of inhibitory functions and future studies should try to disentangle specific psychometrically valid contributors of inhibitory dysfunction. The heightened activity found in inhibitory regions in PD-HS is in agreement with previous evidence in healthy young adults showing greater inhibitory activation when viewing aversive pictures relative to neutral stimuli (Brown et al. 2012). In this context, it is interesting that the medial and superior frontal cortex are interconnected with the ventral striatum, a structure that is considered part of the motivational drive areas, and that is involved in the HS condition. It is tempting to speculate that the observed context-dependent trend for a deactivation of the superior frontal gyrus in the ON, compared to the OFF state (see Fig. 2C, right panel), indicates that context-specific attenuation of inhibitory areas is sensitive to dopaminergic stimulation. This result would be generally in line with DA-induced changes seen in PD with pathological gambling in brain regions that are implicated in inhibitory control (van Eimeren et al. 2009, 2010). This would also be in agreement with Politis et al., who put forward that dopaminergic treatment may decrease sexual inhibition in the cortex, which in turn increases seeking behaviors and contribute to hypersexuality in these patients (Politis et al. 2013). We could not find differences in the perception of sexual versus neutral targets dependent on the inhibitory context. A reason could be the length of the priming stimulus. The neutral or the inhibitory stimuli were presented over 10 s. A previous behavioral study of Andrews et al. presented an inhibitory context as a subliminal stimulus (< 0.5 s) in healthy controls, which suppressed sexual arousal (Andrews et al. 2015). At first, we also tried a contextual framing with a subliminal stimulus in a few PD patients, which did not lead to any effect possibly due to a reduced impetus in PD. It could be possible that the length of the inhibitory stimulus has to be different in every subject to suppress sexual arousal sufficiently. A behavioral paradigm with different lengths of the inhibitory stimulus in each participant prior to the MRI scan could be feasible to titrate an adequate stimulus length that suppresses the reactivity to sexual cues.

## Conclusions

This pilot study showed a greater neuronal activation in PD+HS in brain regions that are associated with erotic content processing. We found an enhanced inhibitory activity in PD-HS group during inhibitory contextual framing, but contrary to our hypothesis, inhibitory contextual framing did not suppress reactivity to sexual cues. In general, the paradigm of this pilot study partially worked, but it should be replicated with a greater cohort.

## Data Availability

The data presented in the present study are available upon reasonable request from the corresponding author.
